# Are the indications for postoperative radiotherapy in the NCCN guidelines for patients with gastric adenocarcinoma too broad? A study based on the SEER database

**DOI:** 10.1186/s12885-018-4957-6

**Published:** 2018-11-03

**Authors:** Ze-Ning Huang, Qi-Yue Chen, Chao-Hui Zheng, Ping Li, Jian-Wei Xie, Jia-Bin Wang, Jian-Xian Lin, Jun Lu, Long-Long Cao, Mi Lin, Ru-Hong Tu, Ju-Li Lin, Hua-long Zheng, Chang-Ming Huang

**Affiliations:** 1 Department of Gastric Surgery, Fujian Medical University Union Hospital, No.29 Xinquan Road, Fuzhou, 350001 Fujian Province China; 2 Department of General Surgery, Fujian Medical University Union Hospital, Fuzhou, China; 3 Key Laboratory of Ministry of Education of Gastrointestinal Cancer, Fujian Medical University, Fuzhou, China; 4 Fujian Key Laboratory of Tumor Microbiology, Fujian Medical University, Fuzhou, China

**Keywords:** Postoperative radiotherapy, Indication, NCCN guidelines, Disease-specific survival rate, Nomogram, Decision curve

## Abstract

**Background:**

The types of patients with gastric adenocarcinoma (GA) for whom postoperative radiotherapy can improve the disease-specific survival rate (DSS) remain controversial. This study aims to explore the ideal indications.

**Methods:**

Patients in the Surveillance, Epidemiology, and End Results (SEER) database with T3–4Nx or TxN+ GA from January 1988 to December 2012 were included and divided into a postoperative chemoradiotherapy group (Group R) and a postoperative chemotherapy group (Group C). We established a nomogram to predict DSS and then divided entire patient cohort into low-risk and high-risk groups based on the DSS predicted by the nomogram.

**Results:**

The Cox multiple regression analysis demonstrated that various risk factors affected DSS for Group R. Based on these risk factors, a nomogram for predicting DSS was established. The decision curve indicated that the best clinical effect could be obtained when the threshold probability was 0–58%. The patients were then divided into low-risk (< 69 points) and high-risk (≥ 69 points) groups according to the five-year DSS predicted. DSS was significantly better for Group R than for Group C for high-risk patients (*P* < 0.001) but was similar for low-risk patients (*P* = 0.732).

**Conclusion:**

At present, the National Comprehensive Cancer Network (NCCN) guidelines may include an overly broad range of indications for postoperative radiotherapy for patients with GA. For intestinal GA patients with a postoperative pathologic stage of T1 N1 who are younger than 65 years, have had more than 15 lymph nodes dissected, and have received postoperative chemotherapy, postoperative radiotherapy should not be recommended.

**Electronic supplementary material:**

The online version of this article (10.1186/s12885-018-4957-6) contains supplementary material, which is available to authorized users.

## Background

Although the morbidity of gastric adenocarcinoma (GA) has declined in recent years, this disease remains the fourth most fatal malignancy in the world [1, 2]. At present, surgery is the only reliable cure for GA [3–5]. Regional recurrence and distant metastasis are the main causes of reduced survival among patients with GA [6, 7]. However, postoperative adjuvant chemoradiotherapy can reduce the risks of regional recurrence and distant metastasis [8–12]. Therefore, the rational use of postoperative chemoradiotherapy can improve patient prognoses. The effects of postoperative adjuvant chemotherapy have been widely recognized. In contrast, although the NCCN guidelines for GA that exhibits mitosis [13] clearly suggest that a postoperative pathological stage of T3–4Nx or TxN+ indicates the feasibility of adjuvant chemoradiotherapy after surgery, which means that postoperative radiotherapy is based on postoperative chemotherapy, a portion of patients who undergo radiotherapy experience severe postoperative complications [14–16], and the curative effects of radiotherapy are influenced by various pathological factors. As a result, the indications for postoperative radiotherapy remain controversial [17]. Thus, the purpose of this study was to use the Surveillance, Epidemiology, and End Results (SEER) database to explore risk factors for prognosis for patients who undergo postoperative radiotherapy; perform stratified analyses of the effects of radiotherapy on postoperative disease-specific survival rate (DSS) for patients with GA; construct a nomogram to filter out patients who are unsuitable for postoperative radiotherapy; and provide a reference for the reasonable implementation of postoperative radiotherapy.

## Methods

### Study population and evaluation parameters

Between January 1988 and December 2012, data were collected from patients with GA of pathological stage T3–4Nx or TxN+ from the SEER database (registration number: 14088-Nov2015). For all cases, tumor stages were reevaluated using the 7th American Joint Committee on Cancer (AJCC) TNM staging system (October 2016) [18].

The inclusion criteria were (1) GA had been confirmed via biopsy; (2) the primary site was limited to the stomach; (3) the patient had undergone gastrectomy; (4) the patient had received postoperative chemoradiotherapy or postoperative chemotherapy; and (5) the pathological stage was T3–4Nx or TxN+. The exclusion criteria were (1) the patient had received preoperative radiotherapy, intraoperative radiotherapy, or radiotherapy and surgery in an uncertain sequence; (2) there was incomplete basic information (including race, gender, and age, among other characteristics) for the patient; (3) distant metastasis had occurred; (4) the pathologic diagnosis was incomplete, and tumor stage could not be accurately assessed; and (5) survival information was unclear. Ultimately, 12,329 cases were included; patients were divided into a postoperative chemoradiotherapy group (Group R, *n* = 7424) and a postoperative chemotherapy group (Group C, *n* = 4905).

Sociodemographic and clinicopathological data were routinely collected. Race was divided into three groups: white, black and other (which included American Indian, Alaskan Native, Asian, and Pacific Islander). Age was divided into two groups (≤ 65 years and > 65 years) in accordance with international standard survival classification categories for age [14]. Using the X-tile program, optimal thresholds for tumor size (based on the longest diameter) were applied to classify the patients into the following groups: < 55 mm, ≥ 55 mm, linitis plastica, and size could not be assessed. Tumor sites were divided into the following four subsite categories: middle and distal third (C16.2, C16.3, and C16.4); proximal third (C16.0 and C16.1); stomach, NOS (C16.5, C16.6, and C16.9); and overlapping (C16.8). The tumors were pathologically categorized as low grade (well and moderately differentiated), high grade (poorly differentiated and undifferentiated) and Gx grade (grade could not be evaluated). With respect to histological type, tumors were categorized as intestinal (8144/3) and other. Variables in the SEER database that have not been mentioned above, such as complications, postoperative complications and incision, were not included in the study. Disease-specific survival time was calculated from the date of surgery until the time of death due to GA or another cause (such as pneumonia or coronary heart disease), and cases for which a follow-up termination event had not yet occurred were excluded from consideration.

### Statistical analysis

Measurement data were examined using chi-square tests or Fisher’s exact test, and enumeration data were analyzed using t-tests or the Mann-Whitney U test. Survival curves were created using the Kaplan-Meier method, and log-rank tests were used to assess between-group differences. The independent risk factors that affected DSS for patients who received radiation were identified using a Cox regression model. A nomogram for predicting DSS was established, and the accuracy of this nomogram was quantified using Harrell’s concordance index (C-index). Internal validation involved the adoption of the calibration curve and 1000 repeats. A plot was produced in which the real line represents actual values and the dotted line represents predicted values. Recursive partitioning was used to choose the most appropriate cutoff point dividing the nomogram to predict 5-year DSS. The analysis process is as follows. The total score of the whole patient cohort is divided into <A, ≥A, two parts, and gradually under different cutoff points (A) to compare the two parts of the patient’s DSS, until the cut-off point that results in the most significant difference between the two parts is identified [19, 20]. In this study, when A = 69, patients with a score < 69 and > 69 showed the most significant difference in DSS (*p* < 0.001). The decision curve verified the application of the nomogram; the Y-axis in the figure represents the benefit of postoperative radiotherapy, the X-axis represents the threshold probability, the horizontal full lines represent the benefit of all the patients that did not receive postoperative radiotherapy, the oblique full line represents the benefit of all the patients receiving postoperative radiotherapy, the diagonal dotted line represents the benefit according to the nomogram scores to decide whether a patient should accept postoperative radiotherapy. The threshold for statistical significance was set at *P* < 0.05. Statistical analyses were performed using SPSS® for Windows® version 19.0 (IBM, Armonk, New York, USA), X-tile and R version 3.2.3 (http://www.r-project.org).

## Results

### Between-group comparisons of overall patient characteristics

Table 1 presents comparisons of general clinical data for the patients in Group R and Group C. There were significant differences between the two groups of patients with respect to gender, race, age, tumor infiltration depth, lymph node metastasis, lymph node number, whether more than 15 lymph nodes were dissected, tumor size, and tumor differentiation grade (*P* < 0.05 for all comparisons). The two groups did not significantly differ with respect to histological type (*P* > 0.05).

**Fig. 1:**
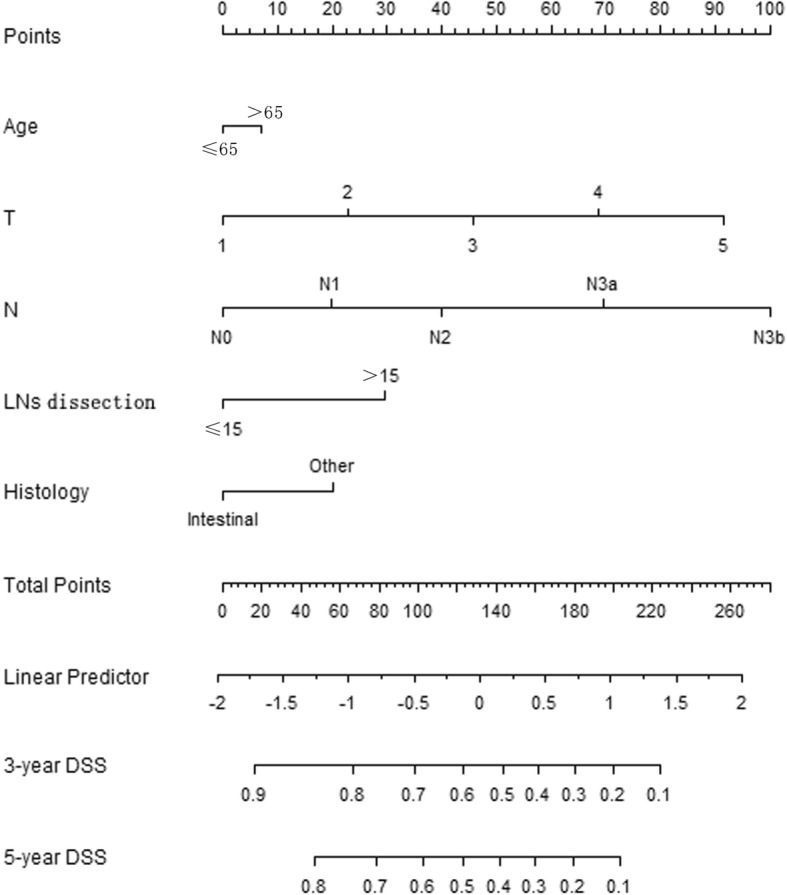
The nomogram for DSS in Group R(C-index = 0.704)

**Table 1:** Demographic and Clinicopathologic Variables of the Development and Validation Cohorts

Variable	Chemoradiotherapy Cohort(*n* = 7424)		Chemotherapy Cohort(*n* = 4905)		
	No. of Patients	%	No. of Patients	%	χ2test P
Sex					.000
Female	2705	36.4	1805	36.8	
Male	4719	63.6	3100	63.2	
Race					.000
Black	1033	13.9	606	12.4	
Other (American Indian/AK Native, Asian/Pacific Islander)	1628	21.9	1007	20.5	
White	4763	64.2	3292	67.1	
Age y					.000
≤ 65	4314	58.1	2656	54.1	
> 65	3110	41.9	2249	35.9	
Depth of invasion					.000
Mucosa/Submucosa	448	6.0	273	5.6	
Proper muscle	622	8.4	321	6.5	
Subserosa	2877	38.8	1858	37.9	
Serosa	2700	36.4	1692	34.5	
Adjacent organ invasion	777	10.5	761	15.5	
Metastatic LNs, No.					.000
0	1033	13.9	866	17.7	
1–2	1883	25.4	1260	25.7	
3–6	2047	27.6	1186	24.2	
7–15	1855	25.0	1096	22.3	
>15	606	8.2	497	10.1	
LNs dissection, No.					.000
>15	3474	53.2	2567	47.7	
≤ 15	3950	46.8	2338	52.3	
Size mm					.000
<55	3670	49.4	2193	44.7	
≥ 55	3144	42.3	2038	41.5	
Size of tumor cannot be assessed	610	8.2	674	13.7	
Primary Site					.000
Proximal third	1590	21.4	1135	23.1	
Mid	2256	30.4	1372	28.0	
Distal third	2335	31.5	1429	29.1	
Stomach, NOS	574	7.7	436	8.9	
Overlapping lesion of stomach	669	9.0	533	10.9	
Grade					
Low	5499	74.1	3632	74.0	
High	1672	22.5	1025	20.9	
Gx	253	3.4	248	5.1	
Histology adenocarcinoma					.751
Other types	6566	88.4	4348	88.6	
Intestinal type	858	11.6	557	11.4	
AJCC 7th staging					.000
Ib	544	7.3	332	6.8	
IIa	673	9.1	559	11.4	
IIb	1308	26.7	785	16.0	
IIIa	2230	30.0	1358	27.7	
IIIb	1883	25.4	1208	24.6	
IIIc	786	10.6	663	13.5	
Follow-up,month					
Median	26		18		
Range	0–318		0–319		

*Abbreviations*:*LN*,lymph node;*No*.,number; *NOS*, not otherwise specified;*AJCC*, American Joint Committee on Cancer;*Gx*, grade could not be evaluated

### The nomogram for DSS in group R

Additional file 1 Table S1 shows results from univariate and multivariate Cox regressions, which were used to predict DSS for Group R. After stepwise backward variable selection, only age, tumor infiltration depth, lymph node metastasis, whether more than 15 lymph nodes were dissected, and histological type (P < 0.05) remained in the final model. The final model served as the basis for a multivariate nomogram (Fig. 1). Nomogram predictions are shown for the 3-year and 5-year time points (C-index = 0.704). Additional file 1: Table S2 shows the cut-off point for risk factors affecting disease-free survival (DSS), and the sum of each factor’s point value is the total score for each patient. The calibration curve (Additional file 2: Figure S1) suggested that the predicted values were consistent with the actual values.

### Stratification of the patients in the two groups by nomogram score

**Fig. 2:**
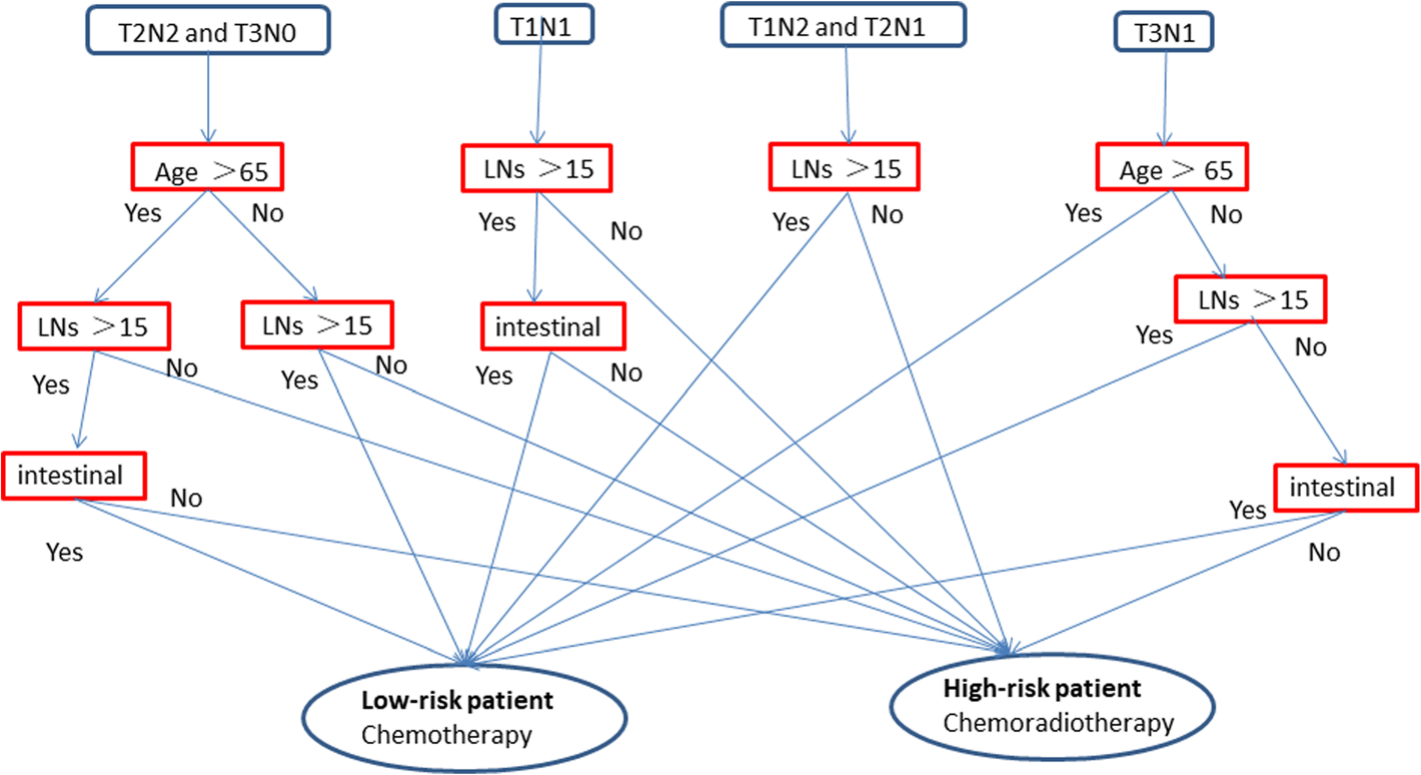
Flow chart of high-risk patients and low-risk patients

In this study, recursive partitioning analysis indicated that the optimal cut-off point for nomogram-predicted 5-year DSS was 69. All of the patients were divided into two groups using this cut-off point, with patients with < 69 and ≥ 69 points categorized as low- and high-risk patients, respectively. Then, the T3–4 Nx and TxN+ patients were combined with those meeting the inclusion criteria, and the flow chart of high-risk patients and low-risk patients is shown in Fig. 2.

**Fig. 3:**
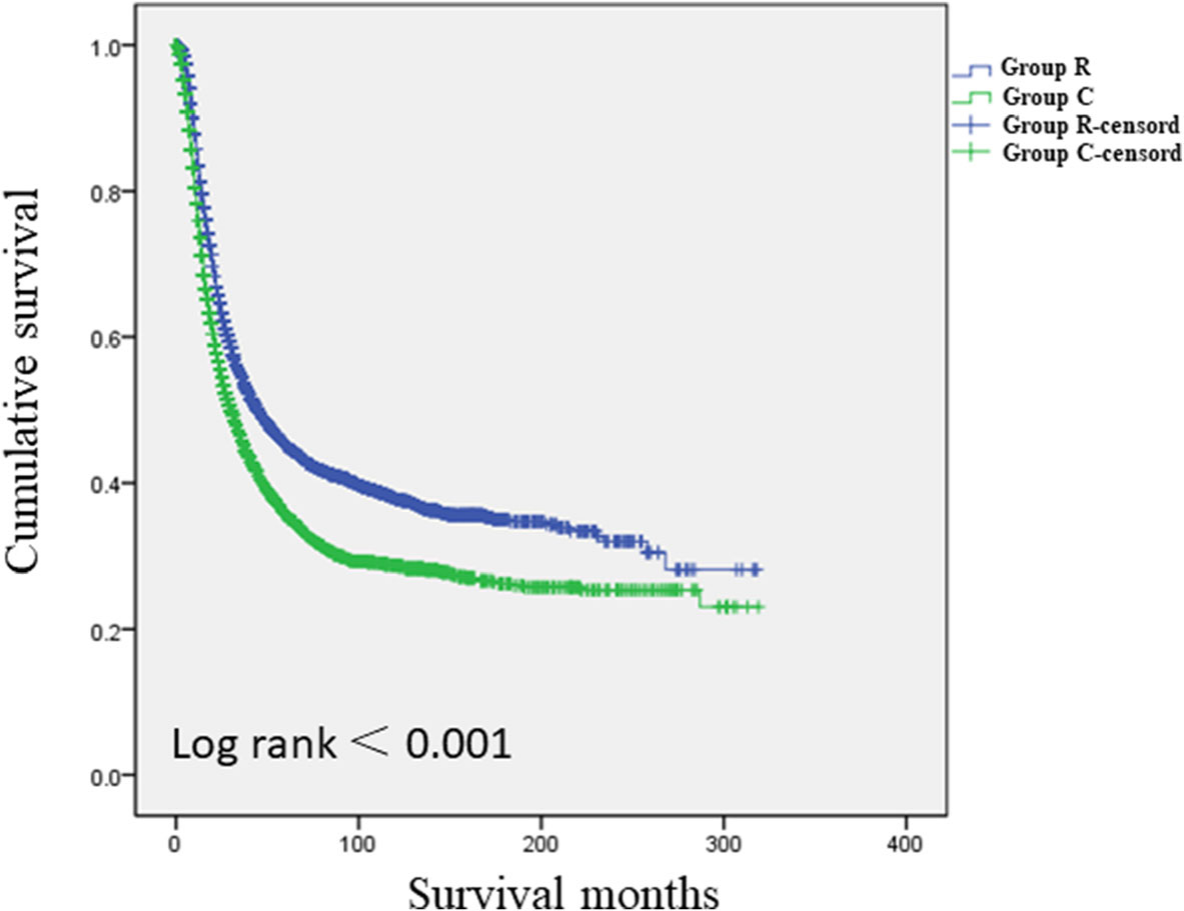
Comparisons of DSSs for the two groups for high-risk patients(Log-rank = 0.732)

### Comparisons of DSSs for the two groups for patients with different risk levels

**Fig. 4:**
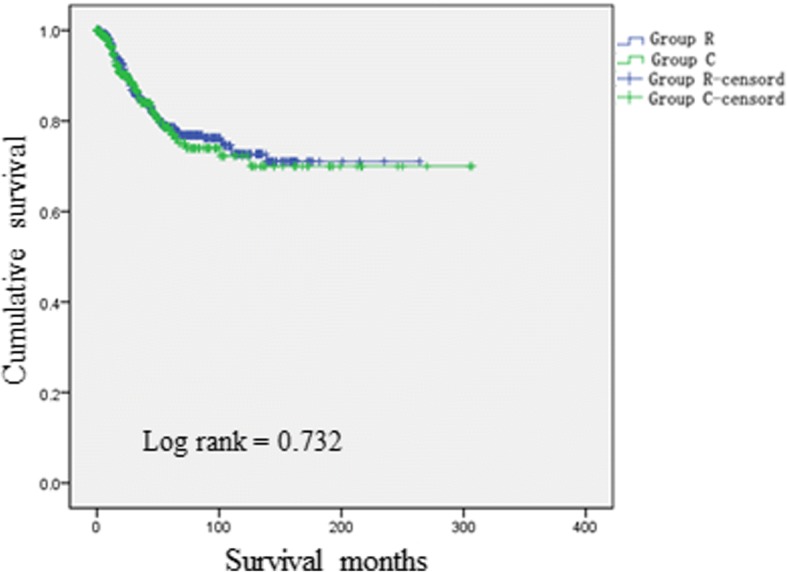
Comparisons of DSSs for the two groups for low-risk patients (Log-rank < 0.001)

Additional file 3 Figure S2 presents a comparison of DSSs for the two groups. DSS was significantly better for Group R than for Group C (log-rank < 0.001). Further stratification analysis revealed that DSS was significantly better for Group R than for Group C for high-risk patients (log-rank < 0.001; Fig. 3) but that the DSSs for the two groups did not significantly differ for low-risk patients (log-rank = 0.732; Fig. 4).

### The decision curve

**Fig. 5:**
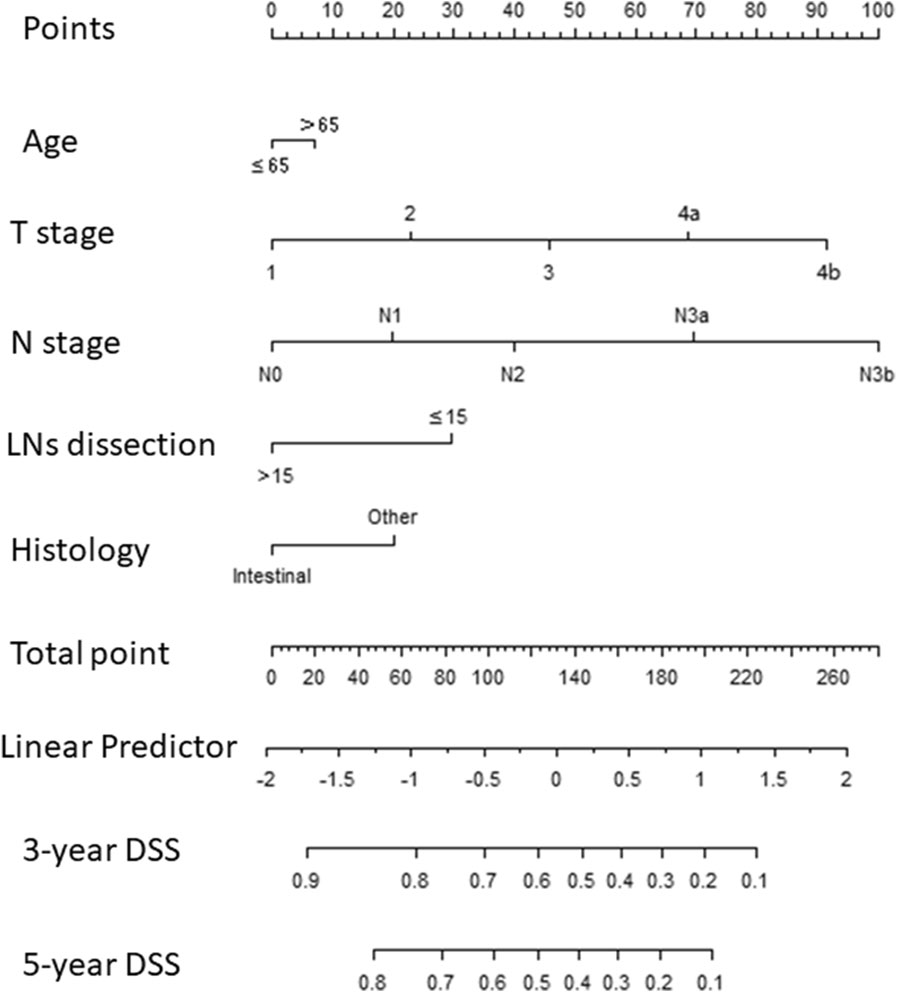
Decision curve based on the nomogram

A decision curve was established based on the nomogram (Fig. 5). The results showed that when the threshold probability is 0–58%, the benefit of the oblique line (whether to administer radiotherapy in the population according to the nomogram score) is greater than that of the horizontal full line (no radiotherapy for the patients) and the oblique full line (provide radiotherapy for the entire population); in other words, the maximum benefit for the patients can be obtained, which means that in cases involving a tumor of pathological stage T3–4Nx or TxN+, postoperative radiation should be administered to patients with scores > 69 points. Compared to strategies in which all patients or no patients receive postoperative radiotherapy, this approach will maximize the curative effects of radiotherapy.

## Discussion

In recent years, the clinical efficacy of postoperative chemoradiotherapy involving postoperative radiotherapy after postoperative chemotherapy has been confirmed for certain gastric cancer patients in randomized controlled trials[14, 21, 22]. However, although indications for postoperative adjuvant chemotherapy have been widely recognized, indications for postoperative adjuvant radiation therapy remain unclear. It is generally believed that the latter indications are influenced not only by TNM stage but also by factors such as the number of lymph nodes dissected and pathological classification [23]. The current NCCN guidelines suggest that gastric cancer patients with pathological staging of T3–4 Nx or TxN + should accept postoperative radiotherapy; these guidelines use TNM stage as the sole determinant of whether postoperative radiotherapy should be performed and thus cannot be applied in clinical contexts. In the present study, the SEER database was used to investigate more accurate decision factors for postoperative radiotherapy and thereby determine stricter indications for this therapeutic modality. The results show that not all these patients require postoperative radiotherapy, and we suggest that high-risk patients should accept postoperative radiotherapy, whereas low-risk patients should not.

Defining risk factors that influence DSS is an approach for studying indications for postoperative radiotherapy. Thus, each of the indicators in this study was an independent risk factor for DSS according to Multiple Cox regression analysis. Prior studies have demonstrated that more advanced TN stage, larger tumor diameter and a tumor pathology classification of diffuse can increase the difficulty of R0 tumor resection and thereby affect patients’ postoperative DSS [24, 25]. Having fewer than 15 lymph nodes dissected can increase the possibility of postoperative lymph node recurrence [26, 27], which also decreases DSS. The results of this study are similar to those of previous studies. Furthermore, we established a nomogram for predicting DSS for patients who underwent postoperative chemoradiotherapy; after grading the two groups of patients, a cut-off for nomogram-predicted 5-year DSS was determined via recursive partitioning analysis. Subsequently, we stratified our analyses of DSS for patients in the two groups based on this cut-off (69 points). The results showed that for all patients, DSS was significantly better for Group R than for Group C. However, among low-risk patients, DSS did not significantly differ for the two groups, indicating that postoperative radiation did not appear to influence DSS for low-risk patients. This result led us to question whether patients in the low-risk group should be required to undergo postoperative radiotherapy. Previously, basic studies have reported that GAs are not sensitive to radiation; normal gastric mucosa cells will already be injured by the time that cancer cells have been exposed to a lethal dose of radiation. Moreover, viscera adjacent to the stomach, such as the liver, pancreas, and other organs, are sensitive to radiation injury. Given the invasiveness of surgery, which causes decreases in patients’ physical strength, additional radiation can often be difficult for patients to tolerate. Therefore, a significant number of patients experience serious complications after postoperative radiotherapy [13–15]. The results of the present study indicate that for low-risk patients, DSS did not improve after postoperative radiotherapy. Therefore, we believe that postoperative radiotherapy is not appropriate for low-risk patients.

This study further verified the value of applying nomograms via decision curves. A decision curve is used as a simple mathematical model in which the loss function [28] is employed to examine the effectiveness of a statistical model for inferring the outcome of an event; such curves have been widely utilized to evaluate the usefulness and benefit of forecasting models [29–32]. The results of this study showed that when the threshold probability is 0–58%, clinicians should make decisions regarding whether radiotherapy should be administered to patients for whom this treatment is suggested by NCCN guidelines. This approach can produce greater beneficial effects than having either all or none of these patients undergo radiotherapy. The threshold probability represents the clinician’s degree of confidence in postoperative radiotherapy. We believe that if a patient is unable to tolerate complications associated with postoperative radiotherapy, the clinician’s confidence in postoperative radiotherapy for patients with GA will be affected. Prior studies [13–15] have indicated that approximately 48–54% of patients with GA are unable to tolerate complications that arise after postoperative radiotherapy; therefore, we believe that clinicians should have approximately 50% confidence that postoperative radiation will benefit patients. This belief is consistent with the scope of application of the decision curve.

This study involved a large sample and long-term follow-up, and the obtained results were verified and validated. Nonetheless, there are a few limitations. First, a retrospective study may inevitably incorporate bias. Second, although SEER data from a single centralized repository offered the opportunity to examine a large sample, the SEER database is missing certain data regarding characteristics that affect outcomes, such as the positive margin rate and postoperative complications. Consequently, the results of this study may be insufficiently accurate. Therefore, to confirm our findings, more rigorous results should be obtained from multi-center, prospective, large-sample clinical trials.

### Conclusion

The nomogram established in this study can be effectively applied to clinical decision-making. Among patients for whom, T3–4 Nx and TxN+ GA patients,NCCN guidelines would recommend postoperative radiotherapy, only the score of patient determined using the nomogram is > 69 should accept postoperative radiotherapy,but for the score < 69 points,postoperative radiotherapy should not be recommended,because there is no benefit for survival but serious complications.

## Additional files

Additional file 1: **Table S1**. Univariate and Multivariate Cox Regression Model for Prediction of Disease-Specific Survival. **Table S2**. The point values for risk factors affecting disease-free survival. (DOCX 30 kb)

Additional file 2: **Figure S1.**Nomogram predicted probability of 5-years DSS (TIF 276 kb)

Additional file 3: **Figure S2.** Comparisons of DSSs for the two groups for all patients (Log-rank < 0.001). (TIF 35 kb)
