# Supplementary material for: Are the indications for postoperative radiotherapy in the NCCN guidelines for patients with gastric adenocarcinoma too broad? A study based on the SEER database
Source: BMC Cancer. 2018 Nov 3;18:1064. doi: 10.1186/s12885-018-4957-6 (PMC6215633; doi:10.1186/s12885-018-4957-6)
Supplement: Supplementary file 1 — Table S1. Univariate and Multivariate Cox Regression Model for Prediction of Disease-Specific Survival. Table S2. The point values for risk factors affecting disease-free survival. (DOCX 30 kb) [file 12885_2018_4957_MOESM1_ESM.docx]

| **Table S1**. Univariate and Multivariate Cox Regression Model for Prediction of Disease-Specific Survival | | | | | | | | | | | | | | | |
| --- | --- | --- | --- | --- | --- | --- | --- | --- | --- | --- | --- | --- | --- | --- | --- |
|  | **Univariate Model** | | | | | **Full Multivariate Model** | | | | | **Reduced Multivariate Model** | | | | |
| **Variable** | **OR** | **95%CI** | | | **P** | **OR** | **95%CI** | | | **P** | **OR** | **95%CI** | | | **P** |
| **Sex** |  |  |  |  |  |  |  |  |  |  |  |  |  |  |  |
| Female | Ref |  |  |  |  | Ref |  |  |  |  |  |  |  |  |  |
| Male | .977 | .900 | - | 1.060 | .572 | .985 | .905 | - | .1072 | .727 |  |  |  |  |  |
| **Race** |  |  |  |  | .739 |  |  |  |  | .121 |  |  |  |  | .131 |
| White | Ref |  |  |  |  | Ref |  |  |  |  | Ref |  |  |  |  |
| Black | 1.151 | 1.012 | - | 1.309 | .698 | .985 | .868 | - | 1.119 | .818 | .988 | .873 | - | 1.119 | .851 |
| Other (American Indian/AK Native, Asian/Pacific Islander) | 1.458 | 1.259 | - | 1.689 | .459 | .904 | .820 | - | .996 | .041 | .907 | .825 | - | .998 | .045 |
| **Age y** |  |  |  |  |  |  |  |  |  |  |  |  |  |  |  |
| ≤65 | Ref |  |  |  |  | Ref |  |  |  |  | Ref |  |  |  |  |
| >65 | 1.458 | 1.259 | - | 1.689 | .000 | 1.426 | 1.228 | - | 1.655 | .000 | 1.428 | 1.231 | - | 1.556 | .000 |
| **Depth of invasion** |  |  |  |  | .000 |  |  |  |  | .001 |  |  |  |  | .001 |
| Mucosa/Submucosa | Ref |  |  |  |  | Ref |  |  |  |  | Ref |  |  |  |  |
| Proper muscle | .914 | .772 | - | 1.083 | .299 | .132 | .738 | - | 1.040 | .132 | .871 | .734 | - | 1.033 | .113 |
| Subserosa | 1.039 | .902 | - | 1.195 | .598 | .965 | .860 | - | 1.155 | .965 | .994 | .859 | - | 1.150 | .932 |
| Serosa | .833 | .718 | - | .965 | .015 | .010 | .699 | - | .953 | .010 | .813 | .698 | - | .949 | .008 |
| Adjacent organ invasion | 1.016 | .833 | - | 1.239 | .876 | .633 | .774 | - | 1.169 | .633 | .944 | .769 | - | 1.158 | .578 |
| **Metastatic LNs, No.** |  |  |  |  | .002 |  |  |  |  | .028 |  |  |  |  | .031 |
| 0 | Ref |  |  |  |  | Ref |  |  |  |  | Ref |  |  |  |  |
| 1-2 | 1.037 | .925 | - | 1.163 | .535 | 1.032 | .914 | - | 1.165 | .612 | 1.031 | .914 | - | 1.163 | .619 |
| 3-6 | 1.052 | .935 | - | 1.184 | .396 | 1.046 | .925 | - | 1.183 | .469 | 1.042 | .923 | - | 1.177 | .507 |
| 7-15  ≥16 | 1.282 | 1.123 | - | 1.464 | .000 | 1.233 | 1.074 | - | 1.416 | .003 | 1.228 | 1.071 | - | 1.409 | .003 |
|  | 1.117 | .888 | - | 1.404 | .346 | 1.062 | .837 | - | 1.348 | .621 | 1.047 | .826 | - | 1.328 | .701 |
| **LNs dissection,No.**  ＞15  ≤15 |  |  |  |  |  |  |  |  |  |  |  |  |  |  |  |
|  | Ref  1.343 |  | | | .000 | Ref  1.214 | 1.114 |  |  |  | Ref  1.220 |  |  | 1.329 | .000 |
|  |  | 1.197 | - | 1.506 |  |  |  | - | 1.322 | .000 |  | 1.121 | - |  |  |
| **Size mm** |  |  |  |  | .025 |  |  |  |  | .107 |  |  |  |  | .126 |
| ＜55 | Ref |  |  |  |  | Ref |  |  |  |  | Ref |  |  |  |  |
| ≥ 55 | 1.072 | .984 | - | 1.167 | .111 | 1.044 | .957 | - | 1.140 | .331 | 1.042 | .955 | - | 1.136 | .359 |
| Size of tumor cannot be assessed | .854 | .723 | - | 1.007 | .061 | .865 | .732 | - | 1.023 | .091 | .870 | .736 | - | 1.028 | .102 |
| **Primary Site** |  |  |  |  | .819 |  |  |  |  | .854 |  |  |  |  |  |
| Proximal third | Ref |  |  |  |  | Ref |  |  |  |  | Ref |  |  |  |  |
| Mid | 1.054 | .941 | - | 1.179 | .363 | 1.039 | .923 | - | 1.169 | .528 |  |  |  |  |  |
| Distal third | 1.038 | .928 | - | 1.162 | .512 | 1.008 | .896 | - | 1.135 | .889 |  |  |  |  |  |
| Stomach, NOS | 1.094 | .916 | - | 1.308 | .320 | 1.055 | .880 | - | 1.266 | .563 |  |  |  |  |  |
| Overlapping lesion of stomach | 1.001 | .845 | - | 1.187 | .989 | .960 | .805 | - | 1.144 | .649 |  |  |  |  |  |
| **Grade** |  |  |  |  | .134 |  |  |  |  | .664 |  |  |  |  |  |
| Low | Ref |  |  |  |  | Ref |  |  |  |  | Ref |  |  |  |  |
| High | 1.044 | 1.044 | - | 1.142 | .343 | 1.000 | .909 | - | 1.099 | .992 |  |  |  |  |  |
| Gx | .824 | .655 | - | 1.038 | .098 | .899 | .713 | - | 1.133 | .368 |  |  |  |  |  |
| **Histology adenocarcinoma** |  |  |  |  |  |  |  |  |  |  |  |  |  |  |  |
| Intestinal type | Ref |  |  |  |  | Ref |  |  |  |  | Ref |  |  |  |  |
| Other types | 1.343 | 1.197 | - | 1.506 | .000 | 1.277 | 1.133 | - | 1.440 | .000 | 1.280 | 1.139 | - | 1.439 | .000 |

| **Table S2**. The point values for risk factors affecting disease-free survival. | |
| --- | --- |
| Factors | Points |
| Age |  |
| ≤65 | 0 |
| ＞65 | 7 |
| T |  |
| 1 | 0 |
| 2 | 23 |
| 3 | 46 |
| 4a | 69 |
| 4b | 92 |
| N |  |
| 0 | 0 |
| 1 | 20 |
| 2 | 40 |
| 3 | 69 |
| LNs dissected |  |
| ＞15 | 0 |
| ≤15 | 30 |
| Histology |  |
| Intestinal | 0 |
| Other | 20 |
