# Supplementary figures and images for: Are the indications for postoperative radiotherapy in the NCCN guidelines for patients with gastric adenocarcinoma too broad? A study based on the SEER database
Source: BMC Cancer. 2018 Nov 3;18:1064. doi: 10.1186/s12885-018-4957-6 (PMC6215633; doi:10.1186/s12885-018-4957-6)

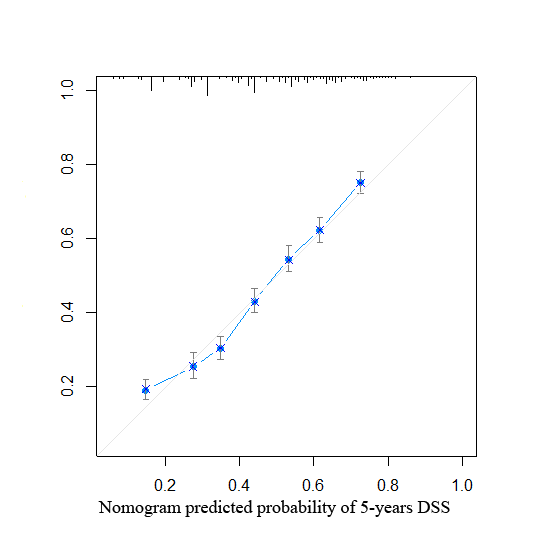

Supplement: Supplementary file 2 — Figure S1.Nomogram predicted probability of 5-years DSS (TIF 276 kb) [file 12885_2018_4957_MOESM2_ESM.tif]

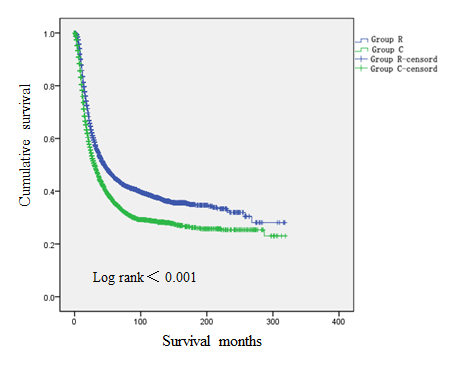

Supplement: Supplementary file 3 — Figure S2. Comparisons of DSSs for the two groups for all patients (Log-rank < 0.001). (TIF 35 kb) [file 12885_2018_4957_MOESM3_ESM.tif]
